# A single pair of pharyngeal neurons functions as a commander to reject high salt in *Drosophila melanogaster*

**DOI:** 10.1101/2023.10.17.562703

**Published:** 2023-10-17

**Authors:** Jiun Sang, Subash Dhakal, Bhanu Shrestha, Dharmendra Kumar Nath, Yunjung Kim, Anindya Ganguly, Craig Montell, Youngseok Lee

**Affiliations:** 1 Department of Bio and Fermentation Convergence Technology, Kookmin University, Seoul, 02707, Republic of Korea; 2 Neuroscience Research Institute and Department of Molecular, Cellular and Developmental Biology, University of California, Santa Barbara, Santa Barbara, CA United States; 3 These authors contributed equally; 4 Lead Contract

## Abstract

Salt is a crucial for survival, while excessive NaCl can be detrimental. In the fruit fly, *Drosophila melanogaster*, an internal taste organ, the pharynx, is a critical gatekeeper impacting the decision to accept or reject a food. Currently, our understanding of the mechanism through which pharyngeal gustatory receptor neurons (GRNs) sense high salt are rudimentary. Here, we found that a member of the ionotropic receptor family, IR60b, is exclusively expressed in a pair of GRNs activated by high salt. Using a two-way choice assay (DrosoX) to measure ingestion, we demonstrate that IR60b and two coreceptors IR25a and IR76b, are required to prevent high salt consumption. Mutants lacking external taste organs but retaining the pharynx exhibit much higher salt avoidance than flies with all taste organs but missing the three IRs. Our findings highlight the critical role for IRs in a pair of pharyngeal GRNs to control ingestion of high salt.

## Introduction

The sense of taste enables animals to find nutritious food while avoiding potentially harmful substances in their environment. Most animals have evolved sophisticated systems to detect and steer clear of consuming levels of substances that are toxic. Salts such as NaCl are vital for a wide array of physiological functions. However, consuming excessive salt can contribute to various health issues in mammals, including hypertension, osteoporosis, gastrointestinal cancer, and autoimmune diseases ^1–6^. Therefore, high concentrations of salt are rejected by most animals.

Multiple studies have delved into how Na^+^ is sensed in the *Drosophila* taste system, shedding light on the mechanisms behind the attraction to low salt and aversion to high salt ^7–14^. The major taste organs in flies, are two bilaterally symmetrical labella, each of which is decorated with 31 gustatory hairs (sensilla). These sensilla are characterized based on size (small, S; intermediate, I; large, L). The I-type sensilla harbor either two gustatory receptor neurons (GRNs), while the S- and L-sensilla contain four. These GRNs fall into five classes (A-E) based on their response profiles. These include A GRNs (formerly sugar GRNs), which respond to attractive compounds such as low salt, sugars, glycerol, fatty acids and carboxylic acids, B GRNs (formerly bitter GRNs), which are activated by high Na^+^, bitter compounds, acids, polyamines, tryptophan, and L-canavanine, ‘C’ GRNs respond to water, ‘D’ GRNs detect high levels of cations such as Na^+^, K^+^ and Ca^2^+, and ‘E’ GRNs sense low Na^+^ levels ^15^.

Several of the 66 member ionotropic receptor (IR) family function in the sensation of low and high salt. These include IR76b and Ir25a, which are IR-coreceptors, and therefore have broad roles in sensing many taste stimuli including low and high salt (sodium) ^8,12,16^, calcium ^17^, several carboxylic acids ^18–20^, fatty acids ^16,21–23^ amino acids ^24^, and carbonation ^25^. A subset of A GRNs, as well as glutamatergic E GRNs are responsible for sensing low salt^8,11,12,15^, and this sensation depends on IR56b working together with the broadly tuned IR25a and IR76b ^8^. Conversely, detection of high salt depends on B GRNs and D GRNs, and IR7c, in conjunction with IR25a and IR76b ^7^. Additionally, two Pickpocket channels, PPK11, PPK19, and Sano have been associated with high salt aversion^26,27^. *ppk19* and *ppk11*, which are members of the pickpocket (*ppk*) gene family, are expressed in taste-sensing terminal organs and play a role in appetitive and aversive behavior in response to low and high salt concentrations, respectively ^27^.

In addition to the labellum and taste hairs on other external structures, fruit flies are endowed with an internal organ in the proboscis, called the pharynx, which functions in the decision to keep feeding or reject a food ^28–32^. The pharynx includes three separate taste organs that line the esophagous: the labral sense organ (LSO), the ventral cibarial sense organ (VCSO), and the dorsal cibarial sense organ (DCSO) ^28,30,31^. Each of these organs include hairless sensilla that house GRNs. A pair of GRNs in the LSO express a member of the gustatory receptor family, *Gr2a*, and knockdown of *Gr2a* in these GRNs impairs the avoidance to slightly aversive levels of Na^+ 14^. Pharyngeal GRNs also promote the aversion to bitter tastants, Cu^2+^, L-canavanine, and bacterial lipopolysaccharides ^33–36^. Other pharyngeal GRNs are stimulated by sugars and contribute to sugar consumption ^28,32,37^. Remarkably, two pharyngeal GRNs function in the rejection rather the acceptance of sucrose ^38^.

In this work, we investigated whether IRs function in the pharynx for avoidance of high Na^+.^ We found that IR60b, along with co-receptors IR25a and IR76b are required in the pharynx for preventing high salt consumption. IR60b is exclusively expressed in a pair of pharyngeal GRNs in the LSO, and these neurons are specifically activated by salt but not by any tested bitter compounds. When we optogenetically activated these IR60b-positive GRNs, proboscis extension responses were inhibited, indicating that these GRNs promote aversive behavior. Moreover, introduction of rat TRPV1 into the IR60b neurons induces aversive towards capsaicin, implying that these IR60b-positive GRNs are essential for instinctive avoidance. To validate the findings, we used a two-way choice DrosoX assay to measure actual ingestion levels. We found that the three *Ir* mutants consumed high salt at levels similar to sucrose over an extended period, emphasizing the critical role of this single pair of pharyngeal GRNs in controlling harmful ingestion of high salt.

## Results and discussion

### *Ir60b* functions in the repulsion to high salt

**Figure 1.**
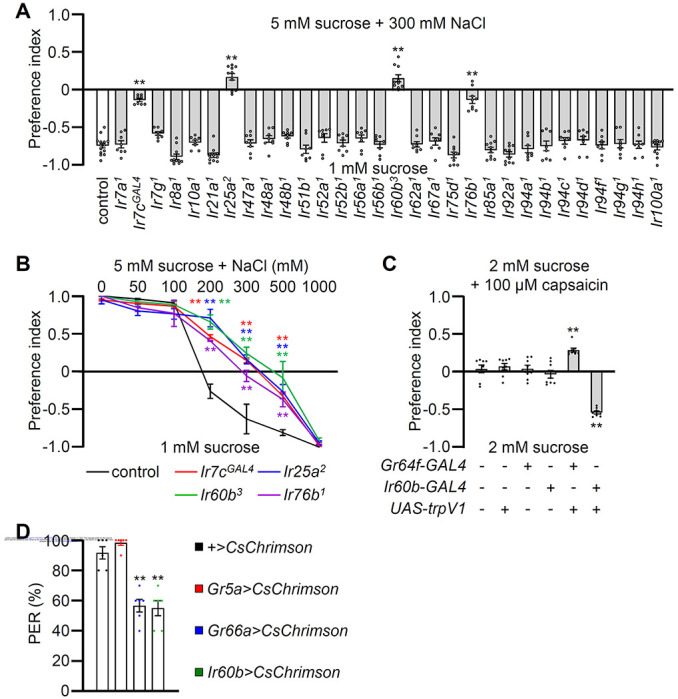
Requirements for four *Ir*s for preferred high salt-containing food and chemogenetic and optogenetic control of *Ir60b*-positive GRNs (A) Binary food choice assay comparing 30 *Ir*-mutants to the control strain (*w^1118^*) for high salt avoidance, n=8–12. (B) Preference of indicated flies observed at various concentrations of NaCl, n=8–12. (C) The gustatory response to the activation of *Gr64f* GRNs or *Ir60b* GRNs by feeding capsaicin to *trpV1*-expressing flies was tested. Binary food choice assays were performed with the indicated flies. The presence or absence of the transgene is indicated by “+” and “−”, respectively. n=8. (D) Optogenetics was employed to measure PER in the indicated flies using red light and sucrose stimulation at the same time. n=6. Multiple sets of data were compared using single-factor ANOVA coupled with Scheffe’s post hoc test. Statistical significance compared with the control was denoted by asterisks (**p < 0.01). All error bars represent the standard error of the mean (SEM).

To identify potential salt sensors in *Drosophila melanogaster*, we conducted binary food choice assays using 30 *Ir* mutants (Figures 1A and S1A). Through screens in which we gave the flies a choice between 2 mM sucrose alone or 2 mM sucrose plus a low, attractive level of salt (50 mM NaCl), we confirmed that *Ir76b* ^12^, *Ir25a*, and *Ir56b* ^8^, are essential for detecting low salt (Figure S1A). Moreover, consistent with Dweck et al. ^8^, using tip recordings to assay tastant-induced action potentials, we found that loss of *Ir56b* nearly eliminated spikes in response to low salt (Figure S1B). Using a *Ir56b-GAL4* to drive *UAS-mCD8::GFP*, we also confirmed that the reporter was restricted to a subset of A GRNs, which were marked tdTomato (Figures S1D—S1F). We generated a *UAS-Ir56b* transgene which restored normal frequencies of action potentials in Ir56b-expressing GRNs (Figure S1B). Moreover, ectopic expression of *UAS-Ir56b* in GRNs that typically have minimal responses to low salt, caused a large increase in salt-induced action potentials (Figure S1C).

In our behavioral screen for *Ir* mutants required for avoiding high salt (300 mM NaCl), we found that in addition to *Ir7c, Ir25a*, and *Ir76b* as previously described ^7^, *Ir60b* was also required (Figure 1A). The *Ir60b* mutant, *Ir60b^3^*, was generated by removing 768 base pairs, which spanned from 44 base pairs upstream of the predicted transcription start site to encompass the coding region for the initial 241 residues of the 577-amino acid protein (Figure S2A-C). Additionally, we verified the impairment in high salt avoidance using *Ir60b^1^*, a gene previously investigated by Joseph et al. (Figure S2D) ^38^. We conducted dose-response behavioral assays using *Ir60b* mutants, as well as *Ir25a, Ir76b* and *Ir7c* and found that all four mutants exhibited significant deficiencies in avoiding salt concentrations ranging from 200 mM to 500 mM (Figure 1B). Nevertheless, all of the mutants exhibited a strong aversion to extremely high salt concentrations, reaching 1000 mM, a level twice as concentrated as that found in the ocean. This extreme condition could potentially trigger the activation of additional pain or alarm neurons, serving as a protective mechanism to prevent potential tissue and organ damage.

### Activation of *Ir60b* neurons inhibits motivation to feed

To investigate whether activation of *Ir60b* neurons induces aversive behavior, we used both chemogenetic and optogenetic approaches. Capsaicin, a ligand for the mammalian TRPV1 channel, does not normally elicit responses in flies (Figure 1C) ^39^. Therefore, we expressed *UAS-trpV1* under the control of the *Ir60b-GAL4*, and presented the flies with a choice between a 2 mM sucrose and a 2 mM sucrose containing 100 mM capsaicin. We found that the transgenic flies actively avoided capsaicin (Figure 1C), whereas expression of TRPV1 in A (sweet) GRNs (*Gr64f-GAL4* and *UAS-trpV1*) induced a preference for capsaicin (Figure 1C). These findings support the idea that the activation of *Ir60b* neurons leads to gustatory avoidance.

**Figure 2.**
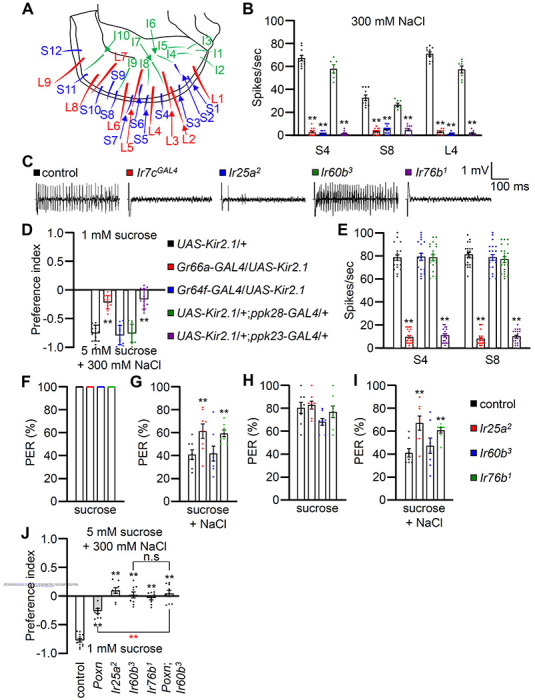
The requirement of pharyngeal *Ir60b*-GRNs in high salt avoidance (A) Schematic representation illustrating the gustatory sensilla arrangement on the fly labellum, following Tanimura’s nomenclature. (B) Tip recording analyses conducted on S4, S8, and L4 sensilla using control, *Ir7c*^*GAL4*^, *Ir25a*^*2*^, *Ir60b*^*3*^, and *Ir76b*^*1*^ strains at 300 mM NaCl, n=10–16. (C) Representative sample traces obtained from S4 sensillum in (D). (D) Binary food choice assays comparing specific GRN-ablated flies to the control strain, with each genotype indicated by different colors. n=12. (E) Tip recording analyses conducted on S4 and S8 sensilla using specific GRN-ablated flies and the control strain at 300 mM NaCl, with each genotype indicated by different colors. n=16–20. (F-I) Proboscis extension reflex (PER) assay was performed using *Ir25a*^*2*^, *Ir60b*^*3*^, *Ir76b*^*1*^, and control strain, n=8–10. (F) PER percentages induced by first 2% sucrose. (G) PER percentages induced by first 2% sucrose with 300 mM NaCl. (H) PER percentages induced by secondary 2% sucrose. (I) PER percentages is induced by secondary 2% sucrose with 300 mM NaCl. (J) Binary food choice assays for 300 mM salt avoidance were conducted with *Poxn* mutant (*Poxn^70-28^/Poxn^ΔM22-B5^*), *Ir25a*^*2*^, *Ir60b*^*3*^, *Ir76b*^*1*^, double mutant (*Poxn^70-28^/Poxn^ΔM22-B5^;Ir60b^3^*), and control strain, n=9-12. All error bars represent SEM. Multiple sets of data were compared using single-factor ANOVA coupled with Scheffe’s post hoc test. Statistical significance compared with the controls or the *Poxn^70-28^/Poxn^ΔM22-B5^* is denoted by black or red asterisks, respectively (**p < 0.01).

To further test the proposal that *Ir60b*-positive GRNs elicit aversive behavior, we expressed CsChrimson, a light-activated cation channel^40^ in *Ir60b* neurons. As controls we drove *UAS-CsChrimson* either in A GRNs (*Gr5a-GAL4*) or B GRNs (*Gr66a-GAL4*). Upon stimulation with red lights and sucrose, nearly all of the control flies (*UAS-CsChrimson* only) or flies expressing *UAS-CsChrimson* in A GRNs extended their proboscis (Figure 2B). In contrast, the PER was notably diminished in flies expressing *UAS-CsChrimson* in B GRNs (*Gr66a-GAL4*) or in *Ir60b* neurons (*Gr66a-GAL4*; 56.7±4.2% and *Ir60b-GAL4*; 55.0±5.0%, respectively; Figure 1D). These results provide compelling evidence supporting the notion that *Ir60b*-positive GRNs induce behavioral aversion.

### *Ir60b* is not required in the labellum to sense high salt

To investigate the physiological responses of labellar sensilla to high salt (300 mM), we conducted tip recordings on each of the 31 sensilla (Figure 2A). Five sensilla, including three S-type (S4, S6, and S8) and two L-type (L4 and L6), exhibited the strongest responses to high salt (Figure S3A). These responses were largely dependent on the broadly tuned IR25a and IR76b, as well as *Ir7c* (Figures 2B, 2C and S3B) as reported^7^. Interestingly, the *Ir60b^3^* deletion mutant did not affect the neuronal responses to high salt in external sensory organs (Figures 2B and 2C). We inactivated individual GRNs by expressing the inwardly rectifying K^+^ channel (*UAS-Kir2.1*) ^41^ in A GRNs (*Gr64f-GAL4*) ^42^, B GRNs (*Gr66a-GAL4*) ^43^, C GRNs (*ppk28-GAL4*) ^44^, and D GRNs (*ppk23-GAL4*) ^17^, and confirmed that the aversive behavior and neuronal responses to high salt primarily relied on B and D GRNs (Figures 2D and 2E) as described ^11^.

To examine the gustatory repulsion to high salt that is mediated through the labellum, we conducted proboscis extension response (PER) assays by stimulating the labellum. Starved control and *Ir* mutant flies extend their proboscis when the labellum is lightly touched by a 100 mM sucrose probe (Figure 2F). Upon a second sucrose offering, the various fly lines exhibited slightly and similarly diminished responses (Figure 2H). When we added 300 mM salt to the sucrose, it significantly reduced the PER in the control group (Figures 2G and 2I; first offering 40.9 ± 4.0%; second offering 41.5 ± 3.7%). Both the *Ir25a^2^* and *Ir76b^1^* mutants also exhibited suppressed PERs, but the suppression was not as great as in the control (Figures 2G and 2I). In contrast, high salt reduced the PER by the *Ir60b^3^* mutant to a similar extent as the control (Figures 2G and 2I; first offering 41.6 ± 6.5%; second offering 47.7 ± 6.7%). This indicates that the labellum of the *Ir60b^3^* detects 300 mM salt normally, even though the mutant is impaired in avoiding high salt in a two-way choice assay (Figure 1A).

### High salt sensor in the pharynx

The observations that *Ir60b* is required for the normal aversion to high salt, but does not appear to function in labellar hairs raises the possibility that *Ir60b* is required in the pharynx for salt repulsion. *Ir60b* is expressed in the pharynx where it plays a role in limiting sucrose consumption ^38^. *Gr2a* is also expressed in the proboscis and contributes to the repulsion to moderate salt levels (150 mM) ^14^. However, the *Gr2a^GAL4^* mutant displays a normal response to high salt (450 mM) ^14^. In our two-way choice assay, which focuses on 300 mM NaCl, we found that salt repulsion displayed by the *Gr2a^GAL4^* mutant was also indistinguishable from the control (Figure S4).

To investigate a role for the pharynx in high salt (300 mM) repulsion, we conducted tests on *Poxn* mutants (*Poxn^70-28^/Poxn^ΔM22-B5^*), in which the external chemosensory sensilla have been converted to mechanosensory sensilla ^45^. As a result, *Poxn* mutants possess only intact internal gustatory organs. We found that the aversive behavior to high salt was reduced in the *Poxn* mutants relative to the control (Figure 2J). However, the diminished avoidance was significantly different from *Poxn^70-28^/Poxn*^*ΔM22*-B5^;*Ir60b^3^* mutants, even though *Poxn^70-28^/Poxn*^*ΔM22*-B5^;*Ir60b^3^* mutants were not significantly different from *Ir60b^3^* in avoidance to high salt (Figure 2J). Furthermore, the *Poxn^70-28^/Poxn^ΔM22-B5^;Ir60b^3^* double mutant exhibited avoidance of high salt at a similar level to *Ir60b^3^*. This suggests that the internal sensor (IR60b-positive GRNs) can override the activation of the labellum in terms of aversive ingestion of high salt. Subsequently, we hypothesized that IR60b might not act as a primary gustatory sensor but rather as a regulator that allows for continued ingestion.

### Quantification of reduced high salt ingestion in *Ir60b* mutants

**Figure 3.**
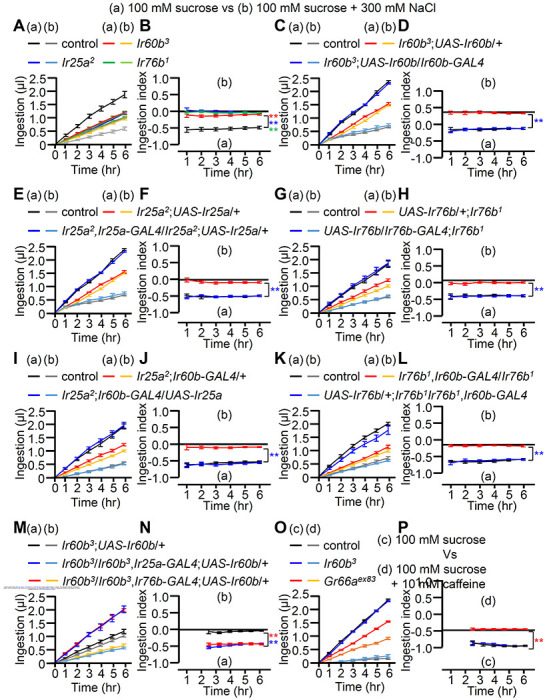
Measurement of food intake utilizing the DrosoX system (A-P) The figures present the ingestion amounts (A, C, E, G, I, K, M and O) and the ingestion index (B, D, F, H, I, J, L, N and P). (A-N) The ingestion of (a)100 mM sucrose alone and (b) in combination with 300 mM NaCl was determined using the Droso-X assay. (A and B) Measurement of food intake with control, *Ir25a*^*2*^, *Ir60b*^*3*^, and *Ir76b*^*1*^, n=12. (C and D) Genetically recovered flies of *Ir60b*^*3*^ driven by *Ir60b-GAL4*, n=12. (E and F) Genetically recovered flies of *Ir25a*^*2*^ driven by *Ir25a-GAL4*, n=12. (G and H) Genetically recovered flies of *Ir76b*^*1*^ driven by *Ir76b-GAL4*, n=12. (I and J) Genetically recovered flies of *Ir25a*^*2*^ driven by *Ir60b-GAL4*, n=12. (K and L) Genetically recovered flies of *Ir76b*^*1*^ driven by *Ir60b-GAL4*, n=12. (M and N) Genetically recovered flies of *Ir60b*^*3*^ driven by *Ir25a-GAL4* and *Ir76b-GAL4* respectively, n=12. (O and P) Measurement of food intake with control, *Ir60b*^*3*^, and *Gr66a*^*ex83*^ at 100 mM sucrose versus 100 mM sucrose with 10 mM caffeine, n=12. All error bars represent SEM. Multiple sets of data were compared using single-factor ANOVA coupled with Scheffe’s post hoc test. Statistical significance compared with the controls (A and P) or each *UAS* only in the mutant condition (D, F, H, J, L and N), is denoted by color asterisks (**p < 0.01).

To assess the ingestion of different food types, we employed the binary food choice assay, a qualitative method that utilizes blue, red, or purple dye colors in the abdomen ^46^. However, for a more precise quantification of food ingestion, we recently developed the DrosoX system (Figure S5A) ^47^. This system allowed us to directly measure the actual amount of food ingested over a period of 6 hours. In these experiments, we present flies with two capillaries: one containing 100 mM sucrose and the other containing 100 mM sucrose mixed with 300 mM NaCl. Control flies exhibited a preference for sucrose-only food, consuming it approximately four times more than the sucrose mixed with salt (Figure 3A). In contrast, the *Ir25a, Ir60b*, and *Ir76b* mutants displayed similar total ingestion levels (Figure 3A) and ingestion volume per hour (Figure 3B; ingestion index) for both tastants. In a prior study, it was observed that *Ir60b* mutant flies consumed high salt at a comparable rate to the control group when the total feeding time was recorded ^38^. However, the DrosoX system now enables us to precisely quantify the ingestion volume. Additionally, we concurrently assessed two distinct tastants and compared their respective consumption levels. Consequently, our approach for evaluating avoidance behavior differs significantly.

To further investigate the requirement for these genes, we performed genetic rescue experiments. We introduced their respective wild-type cDNAs under the control of their cognate *GAL4* drivers, which resulted in a conversion from salt-insensitive behavior to the salt-sensitive behavior observed in wild-type flies (Figures 3C–3H). In addition, the defects in the *Ir25a^2^* and *Ir76b^1^* mutants were fully rescued by expressing the wild-type *Ir25a* and *Ir76b* transgenes, respectively, in the pharynx using the *Ir60b-GAL4* (Figure 3I–3L). This suggests that both IR25a and IR76b act as coreceptors in the IR60b-expressing GRNs. Furthermore, we investigated whether the expression of *UAS-Ir60b* driven by *Ir25a-GAL4* or *Ir76b-GAL4* could rescue the defects observed in *Ir60b^3^*. Remarkably, despite the broad expression of IR60b using these *GAL4* drivers, the *Ir60b* salt ingestion defect was eliminated (Figures 3M and 3N). Thus, it appears that simultaneous activation of GRNs that elicit attractive and aversive salt responses lead to repulsion. This suggests that activation of GRNs that induce salt aversion may suppress GRNs that function in salt attraction. If so, this would be reminiscent of bitter GRNs that suppress sugar GRNs through a GABAergic mechanism ^48^.

Next, we addressed whether IR60b is specifically required for regulating the ingestion high salt. To investigate this, we assessed the consumption of caffeine, strychnine, and coumarin in *Ir60b^3^* flies. We found that *Ir60b^3^* displayed similar consumption patterns to the wild-type control flies for these bitter compounds (Figures 3O, 3P, S5B—S5E). This is in contrast to the impairments exhibited by the *Gr66a^ex83^* mutant (Figures 3O, 3P, S5B—S5E), which is widely required for sensing many bitter chemicals. This indicates that IR60b is involved in regulating the avoidance of high salt ingestion rather than general avoidance responses to toxic compounds. Nevertheless, the role of IR60b in suppressing feeding is not limited to high salt, since IR60b also functions in the pharynx in inhibiting the consumption of sucrose ^38^.

### A single neuron in the LSO depends on *Ir25a, IR60b* and *Ir76b* for responding to both high salt and sucrose

**Figure 4.**
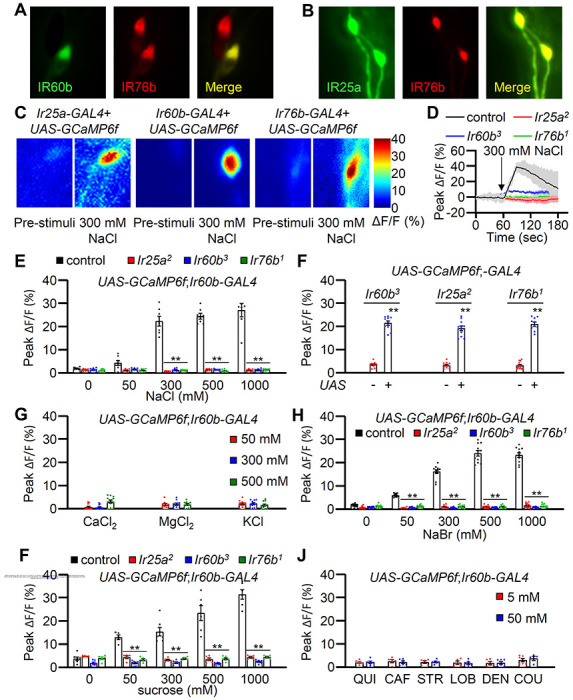
Immunohistochemistry and calcium imaging experiments in the IR60b GRNs (A) Immunohistochemistry was performed using anti-GFP and anti-RFP to visualize the LSO in the pharynx of *UAS-mCD8::GFP/Ir76b-QF2;Ir60b-GAL4/QUAS-tdTomato*. (B) Immunohistochemistry was conducted using anti-GFP and anti-RFP to visualize the LSO in the pharynx of *Ir25a-GAL4/Ir76b-QF2;UAS-mCD8::GFP/QUAS-tdTomato*. (C) Heat map images illustrate changes in GCaMP6f fluorescence before and after stimulation with 300 mM NaCl using the indicated flies. (D) Sample traces depict the responses of *UAS-GCaMP6f;Ir60b-GAL4* to 300 mM NaCl with *Ir25a*^*2*^, *Ir60b*^*3*^, *Ir76b*^*1*^, and control strains. n=10–14. (E) Quantification of *UAS-GCaMP6f;Ir60b-GAL4* responses to various concentrations of NaCl on control, *Ir25a*^*2*^, *Ir60b*^*3*^, and *Ir76b*^*1*^, respectively, n=10–14. (F) Quantification of GCaMP6f responses to 300 mM NaCl on the indicated mutants and rescued flies, n=8–10. The presence or absence of the transgene is indicated by “+” and “−”, respectively. (G) Quantification of *UAS-GCaMP6f;Ir60b-GAL4* responses to 50 mM, 300 mM, and 500 mM of CaCl_2_, MgCl_3_, and KCl on control, n=10–14. (H) Quantification of *UAS-GCaMP6f;Ir60b-GAL4* responses to various concentrations of NaBr on control, *Ir25a*^*2*^, *Ir60b*^*3*^, and *Ir76b*^*1*^, respectively, n=10–14. (I) Quantification of *UAS-GCaMP6f;Ir60b-GAL4* responses to 5 mM and 50 mM concentrations of bitter compounds (quinine, caffeine, strychnine, lobeline, denatonium, and coumarin), n=8–10. (J) Quantification of *UAS-GCaMP6f;Ir60b-GAL4* responses to various concentrations of sucrose on control, *Ir25a*^*2*^, *Ir60b*^*3*^, and *Ir76b*^*1*^, respectively, n=10–14.All error bars represent the SEM. Multiple sets of data were compared using single-factor ANOVA coupled with Scheffe’s post hoc test. Statistical significance compared with the controls is indicated by asterisks (**p < 0.01).

In addition to IR60b, two other broadly required IRs (IR25a and IR76b) also function in repulsion to high salt. Moreover, we found that we could rescue the *Ir25a, Ir60b* or *Ir76b* DrosoX phenotypes using the same *Ir60b-GAL4* to drive expression of the cognate wild-type transgenes in the corresponding mutant backgrounds. These finding imply that all three *Ir*s are coexpressed in the pharynx. Therefore, we examined the relative expression patterns of the *Ir60b-GAL4* reporter with *Ir25a* and *Ir76b* in the pharynx. We observed that the *Ir76b-QF* reporter was expressed in two cells within the labral sensory organ (LSO), one of which colocalized with *Ir60b-GAL4* expression (Figure 4A). Additionally, the expression pattern of *Ir25a-GAL4* perfectly overlapped with that of *Ir76b-QF* in the LSO (Figure 4B). Thus, we suggest that *Ir25a, Ir60b* and *Ir76b* function in the same GRN in the LSO to limit consumption of high salt. We attempted to induce salt activation in the I-type sensilla by ectopically expressing *Ir60b*, similar to what was observed with *Ir56b* ^8^; however, this did not generate a salt receptor (Figures S6A). Thus, the IR25a/IR60a/IR76b channel may require an additional subunit.

To determine whether this GRN in the LSO is activated by high salt, we examined Ca^2+^ responses in the LSO using *UAS-GCaMP6f*, expressed under the control of each *GAL4* driver. In the wild-type LSO, we identified a single cell that responded to 300 mM NaCl (Figure 4C). These data show that the single GRN in the LSO that expresses all three reporters responds to high salt. Moreover, this neuron responded robustly to 300 mM to 1000 mM Na^+^ but not to a low level of Na^+^ (50 mM; Figure 4E). We then examined the Ca^2+^ responses in the *Ir25a^2^*, *Ir60b^3^*, and *Ir76b^1^* mutants, and found that each of them failed to respond to NaCl (Figures 4D and 4E). Additionally, we rescued the deficits in the GCaMP6f responses exhibited by each mutant by expressing a wild-type transgene under control of the corresponding GAL4 driver (Figure 4F). We also tested other Cl^−^ salts (CaCl_2_, MgCl_2_, and KCl) to determine if Cl^−^ rather an Na^+^ induced responses in the IR60b neuron. However, none of these other salts affected these neurons at 50 mM, 300 mM, and 500 mM concentrations tested (Figure 4G). In contrast, NaBr induced GCaMP6f responses (Figure 4H). Thus, the *Ir60b* neuron is responsive to Na^+^ and not Cl^−^. Due to the effects of NaBr on the *Ir60b* neuron, we used the DrosoX assay to determine whether 300 mM NaBr suppressed ingestion of sucrose. We found that the impact of NaBr on sucrose ingestion was similar to that with NaCl (Figures S6B and S6C). We also found that bitter compounds such as quinine, caffeine, strychnine, lobeline, denatonium, and coumarin could not activate *Ir60b* neurons at the concentrations of 5 mM and 50 mM tested (Figure 4I).

It has been shown previously that *Ir60b* is required in single neuron in the pharynx for suppressing sucrose feeding, and this neuron responds to sucrose. ^38^ Therefore, we tested whether the same neuron in the LSO that responds to salt also responds to sucrose. Using GCaMP6f, we found that the *Ir60b* neuron was activated by sucrose in the LSO of control flies, but not in the *Ir25a, Ir60b* and *Ir76b* mutants (Figure 4J). Thus, we conclude that the same LSO neuron depends on the presence of the same three receptors (IR25a, IR60b, and IR76b) for suppressing feeding in response to high salt or to sucrose. Mcdowell et al. demonstrated the presence of IR7c in labellar GRNs sensitive to high salt. They also unveiled the collaborative role of IR7c with IR25a and IR76b in perceiving and responding to high salt concentrations. Consequently, we investigated whether IR7c played a potential role in *Ir60b*-positive pharyngeal GRNs. However, our experiments did not reveal any physiological defects in *Ir7c* mutant flies (Figure S7A). Furthermore, our findings indicated that *Ir7c* is not expressed within the *Ir60b*-positive GRNs (Figure S7B—D).

Although prior research had identified the involvement of IR7c, IR25a, and IR76b in the labellar GRNs for high salt detection, our study introduces a perspective by highlighting the combination of IR25a, IR60b, and IR76b as internal molecular sensors responsible for detecting and ingesting high salt.

## Materials and methods

### Generation of *Ir60b^3^* and *UAS-Ir60b* lines

The *Ir60b^3^* mutant were generated by ends-out homologous recombination ^51^. For generating the construct to injections, approximately two 3-kb genomic fragments were amplified by PCR, and subcloned the DNAs into the pw35 vector of NotI and BamHI sites. Assuming the “A” of the “ATG” starts codon as “+1”, the deleted region was −44 to +724. The construct was injected into *w^1118^* embryos by Best Gene Inc. We outcrossed the mutant with *w^1118^* for 6 generations.

To generate the *UAS-Ir60b* transgenic strain, we employed mRNA to perform reverse transcription polymerase chain reaction (RT-PCR) on the full-length *Ir60b* cDNA, which was subsequently subcloned into the pUAST vector. The insertion took place between the EcoRI and NotI sites designated for *UAS-Ir60b*. The primer set used for amplification is as follows: 5’-GAGAATTCAACTCGAAAATGAGGCGG-3’ and 5’-ATGCGGCCGCAATGCTAATTTTG-3’. The integrity of the cloned cDNA was verified through DNA sequencing. Subsequently, the transformation vector harboring the respective constructs was introduced into *w^1118^* embryos via injection (KDRC).

### Binary food choice assay

We conducted binary food choice assays following the methods outlined in a previous study^46^. Initially, a group of 40–50 flies (3-6 days old) were subjected to an 18-hour starvation period on a 1% agarose substrate. Two mixtures were prepared, each containing a specific dye, and they were distributed in a zigzag pattern. The first mixture consisted of 1% agarose, the indicated concentration of saponin, and 5 mM sucrose with red dye (sulforhodamine B, 0.1 mg/ml). The second mixture contained 1% agarose, 1 mM sucrose, and blue dye (Brilliant Blue FCF, 0.125 mg/ml). The prepared 72-well microtiter dish was then used to transfer the flies, which was placed in a dark and humid chamber. After feeding, the flies were sacrificed by freezing them at −20°C. Subsequently, their abdomen colors were examined under a microscope to identify the presence of red, blue, or purple dye, allowing us to segregate them accordingly. The counts were taken for the number of flies with blue (N_B_), red (N_R_), and purple (N_P_) abdomens. The preference index (P.I) was calculated using the following equation: (N_B_ - N_R_)/(N_R_ + N_B_ + N_P_) or (N_R_ - N_B_)/(N_R_ + N_B_ + N_P_), depending on the specific dye/tastant combinations. A P.I. of −1.0 or 1.0 indicated a complete preference for either 5 mM sucrose with saponin or 1 mM sucrose alone, respectively. A P.I. of 0.0 indicated no preference between the two food alternatives.

### Chemical reagent

Sucrose (CAS No. 57-50-1), tricholine citrate (TCC) (CAS No. 546-63-4), sulforhodamine B (CAS No. 3520-42-1), capsaicin (CAS No. 404-86-4), caffeine (CAS No. 58-08-2), CaCl_2_ dihydrate (CAS No. 10035-04-8), KCl (CAS No. 7447-40-7), quinine (CAS No. 6119-47-7), strychnine (CAS No. 1421-86-9), lobeline (CAS No. 134-63-4), denatonium (CAS No. 6234-33-6), and coumarin (CAS No. 91-64-5) were purchased from Sigma-Aldrich (USA). Brilliant blue FCF (CAS No. 3844-45-9) was purchased from Wako Pure Chemical Industry (Japan). Paraformaldehyde (CAS No. 30525-89-4) was purchased from Electron Microscopy Sciences (USA). NaCl (CAS No. 7647-14-5) was purchased from LPS solution (Korea). NaBr (CAS No. 7647-15-6) was purchased from DUKSAN (Korea). Goat serum, New Zealand Origin was purchased from Gibco (USA).

### Droso-X assay

We conducted Droso-X assays following the methods outlined in a previous study^47^. The amount of ingestion was measured using a Droso-X system (Scitech Korea, Korea) located in a controlled incubator (25°C, 60% humidity). To quantify the ingestion, a mixture comprising 100 mM sucrose and the specified concentration of chemicals was injected into a glass tube (Cat. No. 53432-706; VWR International, USA) using a syringe (KOVAX-SYRINGE 1 ml 26G; KOREA VACCINE, Korea) and needle (Cat No. 90025; Hamilton, Switzerland). Each cuvette contained flies (3-6 days old) and was physically isolated to prevent them from consuming the solution prior to the experiment. The experiment was conducted for a duration of 6 hours, specifically from 9 am to 3 pm. The DROSO X&XD software (Scitech Korea, Korea) was utilized by the Droso-X system to record the amount of solution consumed. The ingestion amount at time X (X h) was calculated as the difference between the initial solution amount (0 h) and the solution amount at time X. The ingestion index (I.I) was calculated in each time point using the following equation: (Ingestion volume_DrosoX_ - Ingestion volume_DrosoXD_)/(Ingestion volume_DrosoX_ + Ingestion volume_DrosoXD_) or (Ingestion volume_DrosoXD_ - Ingestion volume_DrosoX_)/(Ingestion volume_DrosoXD_ + Ingestion volume_DrosoX_), depending on the specific tastant combinations. A I.I. of 0.0 indicated no preference based on their ingestion between the two food alternatives.

### Electrophysiology

Electrophysiology, specifically the tip recording assay, was conducted following the previously described method^52^. The tip recordings were carried out based on Tanimura’s nomenclature. The average frequencies of action potentials (spikes/s) evoked in response are presented, with only spikes occurring between 50 and 550 ms included in the count. For the tip recordings, we followed the established protocol using the specified concentration of saponin dissolved in distilled water with 30 mM tricholine citrate (TCC) for the assay. These electrolytes, 1 mM KCl or 30 mM TCC, served as the recording medium. To begin the recordings, we immobilized flies (3-6 days old) by exposing them to ice. A reference glass electrode filled with Ringer’s solution was inserted through the back thorax and passed into the proboscis. The sensilla on the labial palp were stimulated with a compound dissolved in the buffer solution of the recording pipette, which had a tip diameter of 10-20 μm. The recording electrode was connected to a pre-amplifier (Taste PROBE, Syntech, Germany), which amplified the signals by a factor of 10 using a signal connection interface box (Syntech) and a 100-3,000 Hz band-pass filter. The recorded action potentials were acquired at a sampling rate of 12 kHz and analyzed using Autospike 3.1 software (Syntech). Subsequently, recordings were performed on the indicated sensilla on the labial palp.

### Proboscis extension response assay

The proboscis extension response experiment was conducted with some modifications previously described by a previous study^53^. A group of 20-25 flies (3-6 days old) was deprived of food for 18-20 hours in vials containing wet Kimwipe paper with tap water. After briefly anesthetizing the flies on ice, they were carefully trapped inside a pipette tip with a volume of 20-200 μl. To expose their heads, the edge of the pipette tip was gently cut using a paper cutter blade. The protruded head and proboscis were used to deliver stimuli during the experiment. To stimulate the flies’ tarsi, the head/proboscis and forelegs were extended outside the pipette tip without causing any harm. To eliminate any potential biases due to thirst, water was initially provided to the flies. For both the positive control and initial stimulation, a 2% sucrose solution was used. The tastant stimuli, consisting of either 2% sucrose or 300 mM NaCl, were presented using Kimwipe paper as the medium. To conduct these experiments, we selected flies that responded to sucrose. Flies that did not exhibit a reaction to the sucrose during the initial exposure were excluded from the experiment. The same conditions as the initial exposures were maintained for the second exposure. Each test round involved the use of more than 10 flies.

### Immunohistochemistry

We performed immunohistochemistry as previously described^54^ with slight modifications. The labella of flies (6-8 days old) were dissected and fixed in a solution containing 4% paraformaldehyde (Electron Microscopy Sciences, Cat No 15710) and 0.2% Triton X-100 for 15 minutes at room temperature. After that, the tissues were washed three times with PBST (1x PBS and 0.2% Triton X-100) and then bisected using a razor blade. Subsequently, the tissues were incubated in blocking buffer (0.5% goat serum in 1x PBST) for 30 minutes at room temperature. To detect the target protein, primary antibodies (mouse anti-GFP; Molecular Probes, Cat No A11120; diluted 1:1,000) were added to fresh blocking buffer and left to incubate with the labellum samples overnight at 4 °C. Following this, the tissues were washed three times with PBST and incubated with the secondary antibody (goat anti-mouse Alexa Fluor 488, diluted 1:200) for 4 hours at 4 °C. Afterwards, the tissues were washed three times with PBST and placed in 1.25x PDA mounting buffer (containing 37.5% glycerol, 187.5 mM NaCl, and 62.5 mM Tris pH 8.8). Finally, the samples were visualized using a Leica Stellaris 5 confocal microscope.

### *Ex vivo* calcium imaging

*Ex vivo* Ca^2+^ imaging was performed as previously described^55^ with slight modifications. *Ex-vivo* calcium imaging was conducted using a low melting agarose method. For the experimental process, flies (6-8 days old) expressing *UAS-GCaMP6f* driven by *Ir25a-GAL4, Ir60b-GAL4*, and *Ir76b-GAL4* were used (incubation conditions: humidity: 50-60%, temperature: 25°C, Light/Dark: 12/12 hours). A 0.5% low melting agarose solution was prepared and applied to a confocal dish (Cat No. 102350, SPL LIFE SCIENCE, Korea). A mild swallow deep well was prepared for sample fixation. Subsequently, the heads of the flies were carefully decapitated using sharp razor blades, followed by excising a small portion of the labellum in the extended proboscis region to facilitate tastant access to pharyngeal organs. The prepared tissue sample was then carefully fixed in an inverted position in the pre-prepared well. Videos were recorded with Axio Observer 3 (Carl Zeiss) Adult hemolymph (AHL) composites 108 mM NaCl, 5 mM KCl, 8.2 mM MgCl_3_, 2 mM CaCl_2_, 4 mM NaHCO_3_, 1 mM NaH_2_PO_4_, and 5 mM HEPES pH 7.5. A pre-stimulus solution, AHL was used, followed by the stimulus solution after 60 seconds, enabling direct access of the stimulant with AHL to the pharyngeal neurons. GCaMP6f fluorescence was observed using a fluorescence microscope with a 20x objective, specifically focusing on the relevant area of the pharynx. Videos were recorded at a speed of two frames per second. Neuronal fluorescent activity changes were recorded for 5 minutes following stimulus application. Fiji/ImageJ software (https://fiji.sc) was used to measure fluorescence intensities. A region of interest (ROI) was drawn around the cell bodies, and the Time-Series Analyzer Plugin, developed by Balaji, J. (https://imagej.nih.gov/ij/plugins/time-series.html), was utilized to measure the average intensity for ROIs during each frame. The average pre-stimulation value before chemical stimulation was calculated. ΔF/F (%) was determined using the formula (F_max_-F_0_)/F_0_ × 100%, where F0 represents the baseline value of GCaMP6f averaged for 10 frames immediately before stimulus application, and Fmax is the maximum fluorescence value observed after stimulus delivery.

### Statistical analysis

The error bars on the graph indicate the standard error of the means (SEMs), while the dots represent the number of trials conducted for the experiment. To compare multiple datasets, we utilized single-factor ANOVA coupled with Scheffe’s analysis as a *post hoc* test. Pairwise comparisons were conducted using Student’s t-test. Statistical significance is denoted by asterisks (*p < 0.05, **p < 0.01). We performed all statistical analyses using Origin Pro 8 for Windows (ver. 8.0932; Origin Lab Corporation, USA).

## Figures and Tables

**Table:** Key resources table

Reagent type	Designation	SOURCE	IDENTIFIER
Antibody	Mouse anti-GFP	Molecular probes	Cat # A11120; RPID: AB_221568
Antibody	Rabbit anti-DsRed	Clontech	Cat # 632496; RPID: AB_10013483
Antibody	Goat Anti-mouse Alexa Fluro 488	Invitrogen	Cat # A32723; RRID: AB_2633275
Antibody	Goat anti-rabbit Alexa Fluor 568	Invitrogen	Cat # A11011; RPID: AB_143157
Chemical compound, drug	Sucrose	Sigma-Aldrich	Cat # 9378S
Chemical compound, drug	Tricholine citrate	Sigma-Aldrich	Cat # T0252
Chemical compound, drug	Sulforhodamine B	Sigma-Aldrich	Cat # 230162
Chemical compound, drug	Capsaicin	Sigma-Aldrich	Cat # M2028
Chemical compound, drug	Caffeine	Sigma-Aldrich	Cat # C02750
Chemical compound, drug	CaCl_2_ dihydrate	Sigma-Aldrich	Cat # C3881
Chemical compound, drug	KCl	Sigma-Aldrich	Cat # P9541
Chemical compound, drug	Quinine	Sigma-Aldrich	Cat # Q1125
Chemical compound, drug	Strychnine	Sigma-Aldrich	Cat # S8753
Chemical compound, drug	Lobeline	Sigma-Aldrich	Cat # 141879
Chemical compound, drug	Denatonium	Sigma-Aldrich	Cat # D5765
Chemical compound, drug	Coumarin	Sigma-Aldrich	Cat # C4261
Chemical compound, drug	Brilliant blue FCF	Wako Pure Chemical Industry	Cat # 027-12842
Chemical compound, drug	Paraformaldehyde	Electron Microscopy Sciences	Cat # 15710
Chemical compound, drug	NaCl	LPS solution	Cat # NACL01
Chemical compound, drug	MgCl_2_ hexahydrate	SAMCHUN	Cat # M0038
Chemical compound, drug	NaBr	DUKSAN	Cat # S2531
Chemical compound, drug	Goat Serum, New Zealand origin	Gibco	Cat # 16210064
Genetic reagent (*Drosophila melanogaster*)	*w^1118^*	Bloomington *Drosophila* Stock Center	BDSC:5905
Genetic reagent (*Drosophila melanogaster*)	*Ir7a^1^*	Dr. Y. Lee	^20^
Genetic reagent (*Drosophila melanogaster*)	*Ir7g^1^*: y^1^w*Mi{y^+mDint2^=MIC}Ir7g^MI06687^	Bloomington *Drosophila* Stock Center	BDSC:42420
Genetic reagent (*Drosophila melanogaster*)	*IR7c^GAL4^*	Dr. M. D. Gordon	^7^
Genetic reagent (*Drosophila melanogaster*)	*Ir8a^1^*: w*TI{w[+m*]=TI}Ir8a^1^;Bl^1^L^2^/CyO	Bloomington *Drosophila* Stock Center	BDSC:23842
Genetic reagent (*Drosophila melanogaster*)	*Ir10a^1^*: *w^1118^*Mi{GFP^E.3xP3^=ET1}Ir10a^MB03273^	Bloomington *Drosophila* Stock Center	BDSC:41744
Genetic reagent (*Drosophila melanogaster*)	*Ir21a^1^*: *w^1118^*;PBac{w^+mC^=PB}Ir21a^c02720^	Bloomington *Drosophila* Stock Center	BDSC: *10975*
Genetic reagent (*Drosophila melanogaster*)	*Ir25a^2^*	Dr. L. Voshall	^49^
Genetic reagent (*Drosophila melanogaster*)	*Ir47a^1^*	Dr. Y. Lee	^20^
Genetic reagent (*Drosophila melanogaster*)	*Ir48a^1^*: *w^1118^*;Mi{GFP^E.3xP3^=ET1}Ir48a^MB09217^	Bloomington *Drosophila* Stock Center	BDSC:26453
Genetic reagent (*Drosophila melanogaster*)	*Ir48b^1^*: *w^1118^*;Mi{GFP^E.3xP3^=ET1}Ir48b^MB02315^	Bloomington *Drosophila* Stock Center	BDSC:23473
Genetic reagent (*Drosophila melanogaster*)	*Ir51b^1^*: *w^1118^*;PBac{w^+mC^=PB}row^c00387^ Ir51b^c00387^	Bloomington *Drosophila* Stock Center	BDSC:10046
Genetic reagent (*Drosophila melanogaster*)	*Ir52a^1^*	Dr. Y. Lee	^20^
Genetic reagent (*Drosophila melanogaster*)	*Ir52b^1^*: *w^1118^*;Mi{GFP^E.3xP3^=ET1}Ir52b^MB02231^/SM6a	Bloomington *Drosophila* Stock Center	BDSC:25212
Genetic reagent (*Drosophila melanogaster*)	*Ir52c^1^*: *w^1118^*;Mi{GFP^E.3xP3^=ET1}Ir52c^MB04402^	Bloomington *Drosophila* Stock Center	BDSC:24580
Genetic reagent (*Drosophila melanogaster*)	*Ir56a^1^*	Dr. Y. Lee	^20^
Genetic reagent (*Drosophila melanogaster*)	*Ir56b^1^*: *w^1118^*;Mi{GFP^E3xP3^=ET1}Ir56b^MB09950^	Bloomington *Drosophila* Stock Center	BDSC:27818
Genetic reagent (*Drosophila melanogaster*)	Ir56d^1^: w*;Ir56d^1^	Bloomington *Drosophila* Stock Center	BDSC:81249
Genetic reagent (*Drosophila melanogaster*)	*Ir60b^3^*	Dr. Y. Lee	In this study
Genetic reagent (*Drosophila melanogaster*)	*Ir62a^1^*: y^1^w*;Mi{y^+mDint2^=MIC}Ir62a^MI00895^Iml1^MI00895^/TM3, Sb^1^ Ser^1^	Bloomington *Drosophila* Stock Center	BDSC:32713
Genetic reagent (*Drosophila melanogaster*)	*Ir67a^1^*: y^1^w*;Mi{y^+mDint2^=MIC}Ir67a^MI11288^	Bloomington *Drosophila* Stock Center	BDSC:56583
Genetic reagent (*Drosophila melanogaster*)	*Ir75d^1^*: *w^1118^*;Mi{GFP^E.3xP3^=ET1}Ir75d^MB04616^	Bloomington *Drosophila* Stock Center	BDSC: *24205*
Genetic reagent (*Drosophila melanogaster*)	*Ir76b^1^*	Dr. C. Montell	^12^
Genetic reagent (*Drosophila melanogaster*)	*Ir85a^1^*: *w^1118^*;Mi{GFP^E.3xP3^=ET1}Ir85a^MB04613^Pif1A^MB04613^	Bloomington *Drosophila* Stock Center	BDSC: *24590*
Genetic reagent (*Drosophila melanogaster*)	*Ir92a^1^*: *w^1118^*;Mi{GFP^E.3xP3^=ET1}Ir92a^MB03705^	Bloomington *Drosophila* Stock Center	BDSC: *23638*
Genetic reagent (*Drosophila melanogaster*)	*Ir94a^1^*	Dr. Y. Lee	^20^
Genetic reagent (*Drosophila melanogaster*)	*Ir94b^1^*: *w^1118^*;Mi{GFP^E3xP3^=ET1}Ir94b^MB02190^	Bloomington *Drosophila* Stock Center	BDSC: *23424*
Genetic reagent (*Drosophila melanogaster*)	*Ir94c^1^*	Dr. Y. Lee	^20^
Genetic reagent (*Drosophila melanogaster*)	*Ir94d^1^*:y^1^w*;Mi{y^+mDint2^=MIC}Ir94d^MI01659^CG17380^MI01659^	Bloomington *Drosophila* Stock Center	BDSC:33132
Genetic reagent (*Drosophila melanogaster*)	*Ir94f^1^*: y^1^w*;Mi{y^+mDint2^=MIC}Ir94f^MI00928^	Bloomington *Drosophila* Stock Center	BDSC:33095
Genetic reagent (*Drosophila melanogaster*)	*Ir94g^1^*: *w^1118^*;Mi{GFP^E3xP3^=ET1}Ir94g^MB07445^	Bloomington *Drosophila* Stock Center	BDSC:25551
Genetic reagent (*Drosophila melanogaster*)	*Ir94h^1^*	Dr. Y. Lee	^20^
Genetic reagent (*Drosophila melanogaster*)	*Ir100a^1^*: *w^1118^*;P{w^+mC^=EP}Ir100a^G1984b^ CG42233^G19846^	Bloomington *Drosophila* Stock Center	BDSC: *31853*
Genetic reagent (*Drosophila melanogaster*)	*UAS-mCD8::GFP*	Bloomington Drosophila Stock Center	BDSC:5137
Genetic reagent (*Drosophila melanogaster*)	*UAS-mCD8::GFP*	Bloomington Drosophila Stock Center	BDSC:32184
Genetic reagent (*Drosophila melanogaster*)	*UAS-Kir2.1*	Bloomington *Drosophila* Stock Center	BDSC: *6595*
Genetic reagent (*Drosophila melanogaster*)	*UAS-Ir25a*	Dr. Y. Lee	^17^
Genetic reagent (*Drosophila melanogaster*)	*UAS-Ir60b*	Dr. Y. Lee	In this study
Genetic reagent (*Drosophila melanogaster*)	*UAS-Ir76b*	Dr. C. Montell	^12^
Genetic reagent (*Drosophila melanogaster*)	*Ir25a-GAL4*	Dr. L. Vosshall	^49^
Genetic reagent (*Drosophila melanogaster*)	*Ir60b-GAL4*	Dr. C. Montell	^38^
Genetic reagent (*Drosophila melanogaster*)	*Ir76b-GAL4*	Dr. C. Montell	^12^
Genetic reagent (*Drosophila melanogaster*)	*ppk23-GAL4*	Dr. K. Scott	^50^
Genetic reagent (*Drosophila melanogaster*)	*ppk28-GAL4*	Dr. H. Amrein	^44^
Genetic reagent (*Drosophila melanogaster*)	*Gr66a-GAL4*	Dr. H. Amrein	^43^
Genetic reagent (*Drosophila melanogaster*)	*Gr64f-GAL4*	Dr. A. Dahanukar	^17^
Genetic reagent (*Drosophila melanogaster*)	*Ir76b-QF*	Bloomington *Drosophila* Stock Center	BDSC:51312
Genetic reagent (*Drosophila melanogaster*)	*QUAS-tdTomato: y^1^w^1118^*; P{QUAS-mtdTo mato-3xHA}26	Bloomington *Drosophila* Stock Center	BDSC:30005
Genetic reagent (*Drosophila melanogaster*)	*Poxn^ΔM22-B5^*: y^1^w^67c23^; Mi{ET1}Poxn^MB00113^	Bloomington *Drosophila* Stock Center	BDSC:22701
Genetic reagent (*Drosophila melanogaster*)	*Poxn^70-28^*: *Poxn^70^*/CyO; twi-gal4, UAS-2XE GFP	Bloomington *Drosophila* Stock Center	BDSC:60688
Software	Origin Pro Version	Dr. Y. Lee	https:/www.originlab.com
Software	GraphPad Prism	Dr. Y. Lee	https:/www.graphpd.com
Software	Autospike 3.1 software	Dr. Y. Lee	https://www.syntech.co.za/
Software	Fiji/ImageJ software	Dr. Y. Lee	https://fiji.sc
Software	ZEN lite 2.5 blue	Dr. Y. Lee	https://www.zeiss.com/

## Data Availability

Source data for all figures contained in the manuscript and SI have been deposited in ‘fiigshare’ (https://doi.org/10.6084/m9.figshare.23939394).
